# Supplementary material for: Effect of a retinoic acid analogue on BMP-driven pluripotent stem cell chondrogenesis
Source: Sci Rep. 2024 Feb 1;14:2696. doi: 10.1038/s41598-024-52362-3 (PMC10834951; doi:10.1038/s41598-024-52362-3)
Supplement: Supplementary file 1 [file 41598_2024_52362_MOESM1_ESM.pdf]

Supplemental Information

Effect of a retinoic acid analogue on BMP-driven pluripotent stem cell chondrogenesis.

Fabrizio E. Mancini[1, 2a], Paul E.A. Humphreys[1a], Steven Woods[1], Nicola Bates[1], Sara Cuvertino[3], Julieta O'Flaherty[1], Leela Biant[1], Marco A.N. Domingos[2], Susan J. Kimber[1*]

[1]Division of Cell Matrix & Regenerative Medicine, School of Biological Sciences, Faculty of Biology Medicine and Health, University of Manchester, Oxford Rd, M13 9PT, Manchester, UK

[2]Department of Mechanical, Aerospace and Civil Engineering, School of Engineering, Faculty of Science and Engineering & Henry Royce Institute, University of Manchester, Oxford Rd, M13 9PL, Manchester, UK

[3]Division of Evolution, Infection and Genomics, Faculty of Biology, Medicine and Health, The University of Manchester, Oxford Rd, M13 9PT, Manchester, UK

[a]These authors contributed equally to this work.

*Corresponding Author: Prof Susan J. Kimber. Email: sue.kimber@manchester.ac.uk

List of Supporting Information

Supplementary Figures

Supplementary Figure 1: Quantification of BMP2 batch and manufacturer activity in TC28a2-BRE/P cells.

Supplementary Figure 2 - Cell proliferation of MAN13 hESCs during differentiation induced by the RAPID-E protocol.

Supplementary Figure 3 - RT-qPCR analysis comparing chondrogenic markers expression for hiPSCs differentiated using the RAPID and the RAPID-E protocols.

Supplementary Figure 4 - Hierarchical clustering analysis of all samples comparing the top ~10,000 most highly expressed genes that exhibited at least 2-fold change between two or more samples.

Supplementary Figure 5 –Analysis of transcriptional changes induced by TTNPB addition

Supplementary Figure 6 - Flow cytometry analysis for the limb-bud mesenchyme marker PRRX1 in MAN13 hESCs differentiated by the RAPID-E protocol.

Supplementary Figure 7 - Analysis of HOX gene expression influenced by TTNPB addition.

Supplementary Figure 8 - Gene expression of articular and hypertrophic cartilage markers.

Supplementary Figure 9 – Analysis of top 50 most expressed genes associated with "Cell adhesion" (GO:0098602) at day 6 and day 11

Supplementary Tables

Supplementary Table 1 – Overview of the RAPID-E differentiation protocol – For allowing pellet formation, TTNPB supplementation was ceased at day 10.

Supplementary Table 2 - Overview of antibodies used for immunohistochemistry and the concentration used.

Supplementary Table 3 – Primers used for RT-qPCR

Supplementary Figures

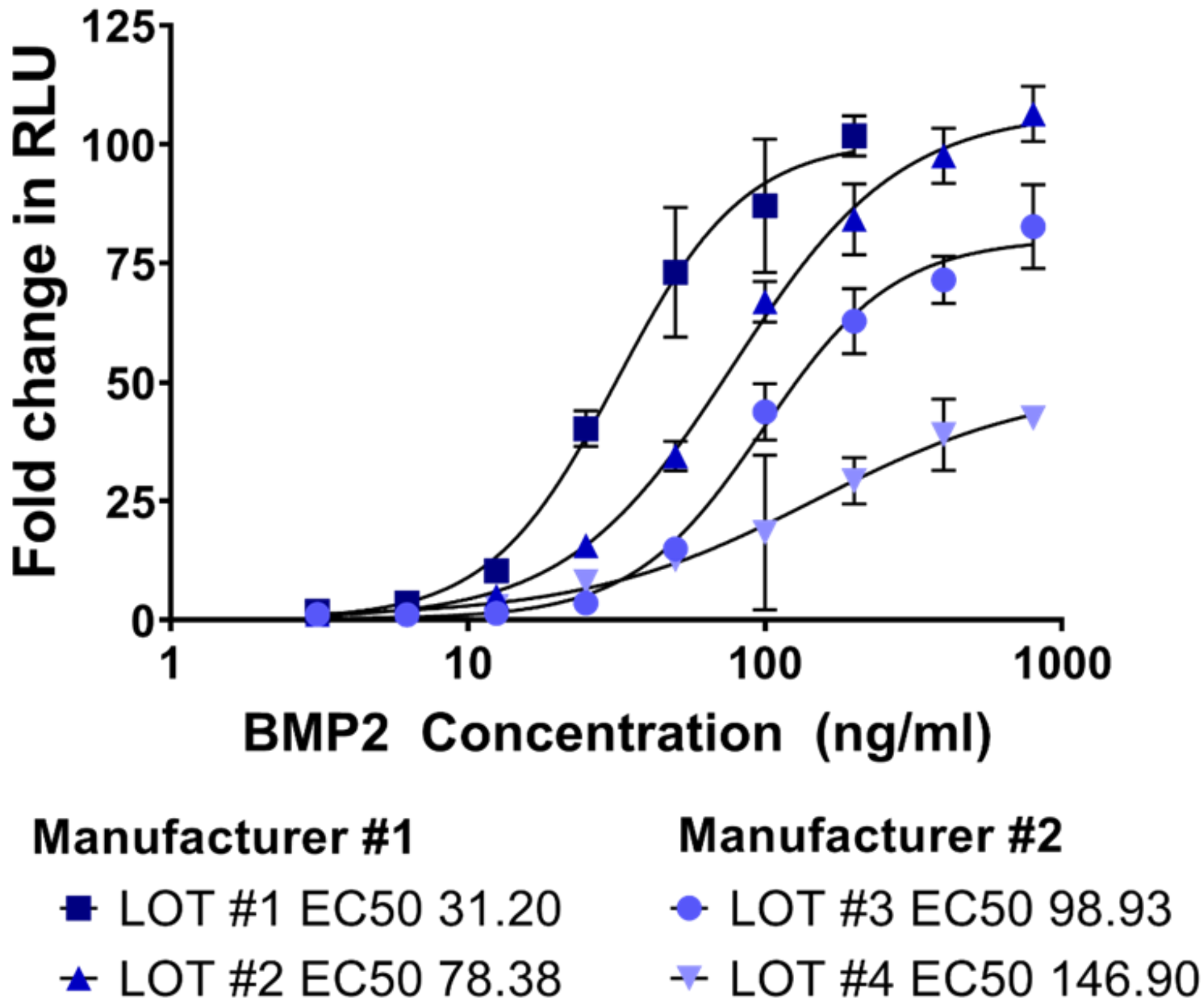

Supplementary Figure 1 - Quantification of BMP2 batch and manufacturer activity in TC28a2-BRE/P cells. Data presented as mean fold change in RLU from unstimulated cells ± SD. n = 3 wells per condition.

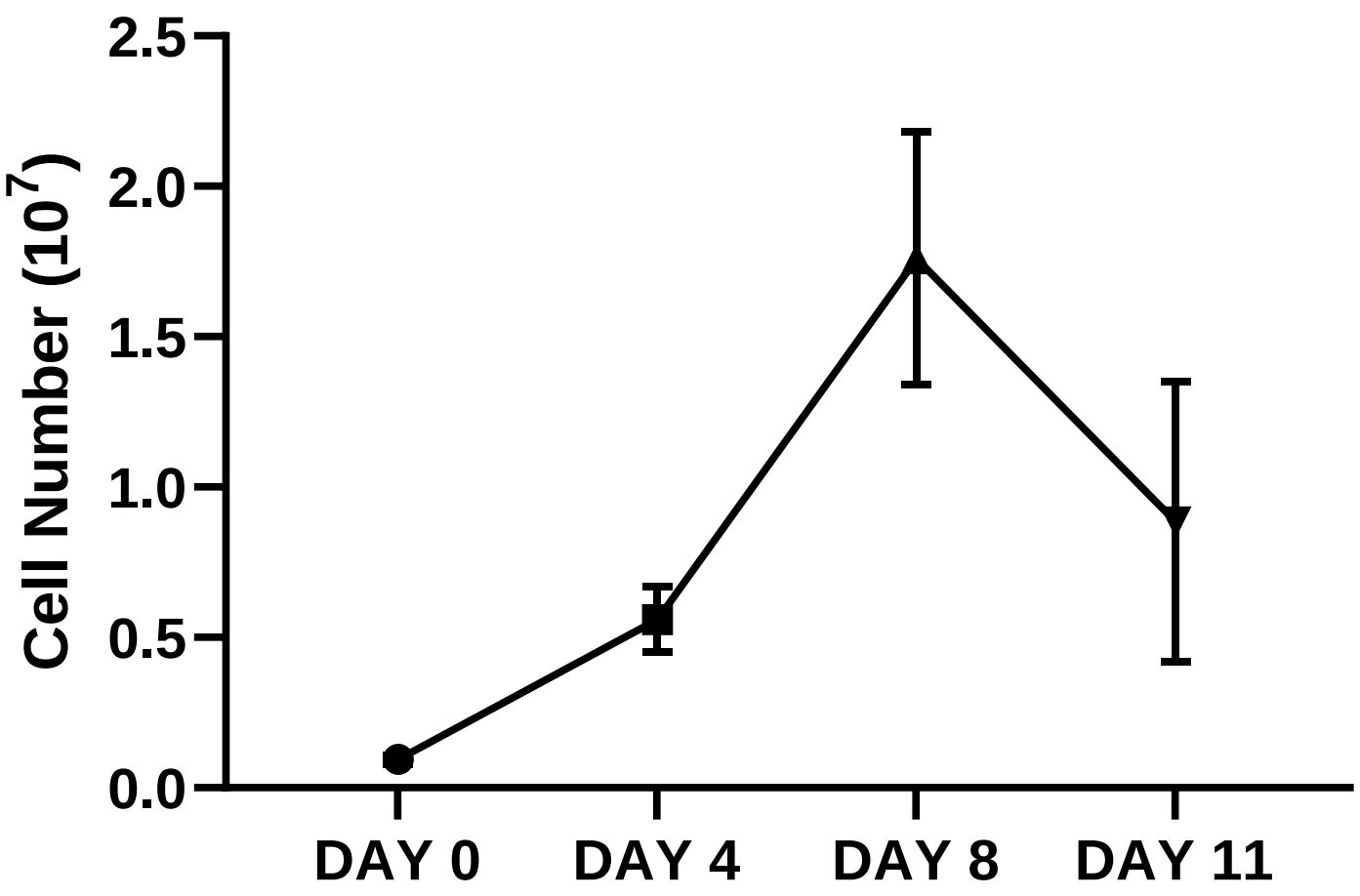

Supplementary Figure 2 - Cell proliferation of MAN13 hESCs during differentiation induced by the RAPID-E protocol. Data are presented as means ± SEM from N=2 independent experiments.

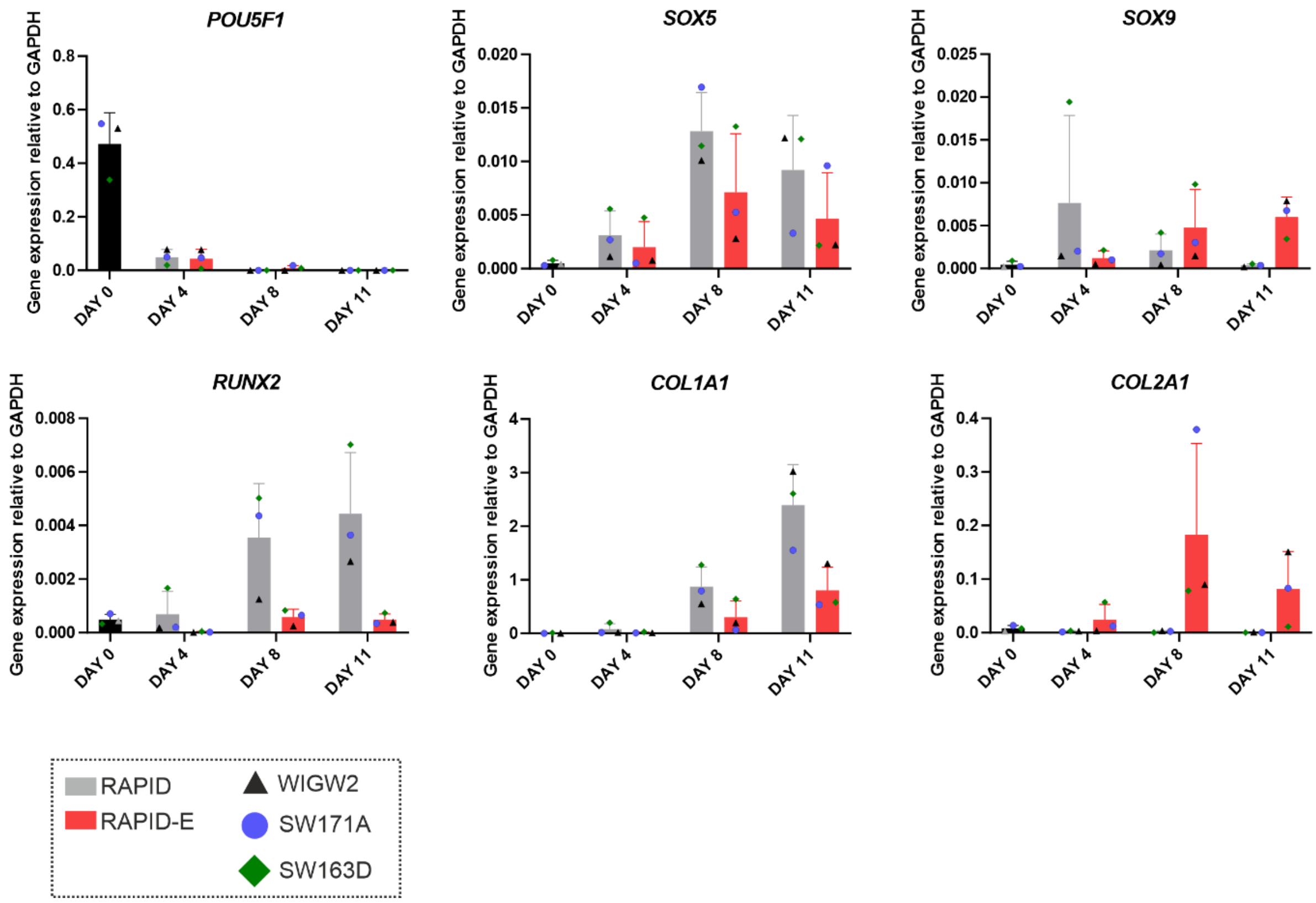

Supplementary Figure 3 - RT-qPCR analysis comparing chondrogenic markers expression for hiPSCs differentiated using the RAPID and the RAPID-E protocols. Data presented as individual values of 3 independent biological repeats using different hiPSCs lines: SW171A, SW163D, and WIGW2. Bars represent the mean across hiPSC lines and error bars represent SEM.

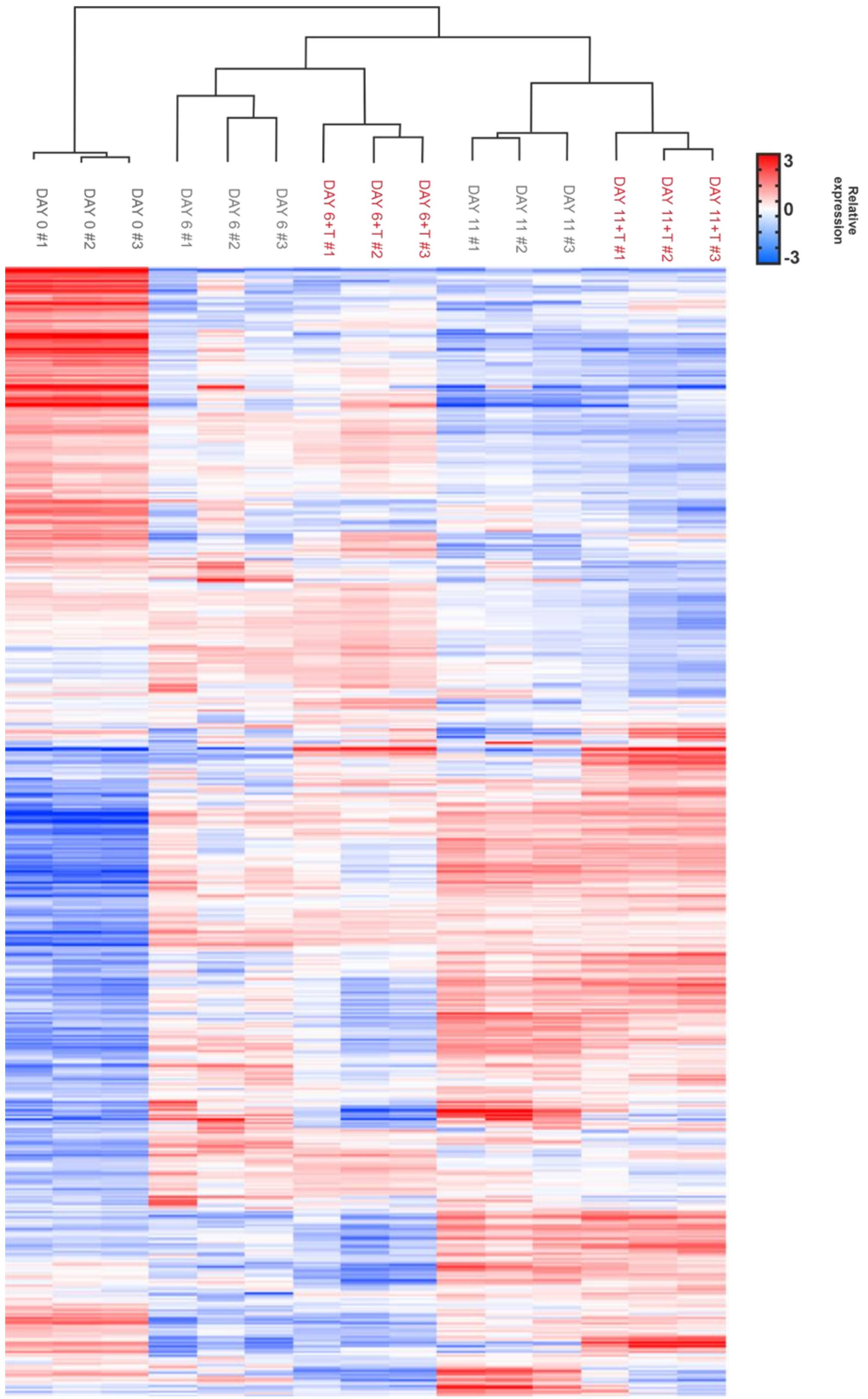

Supplementary Figure 4 - Hierarchical clustering analysis of all samples comparing the top ~10,000 most highly expressed genes that exhibited at least 2-fold change between two or more samples. Clustering and visualisation performed using Cluster 3.0 and Treeview software (31).

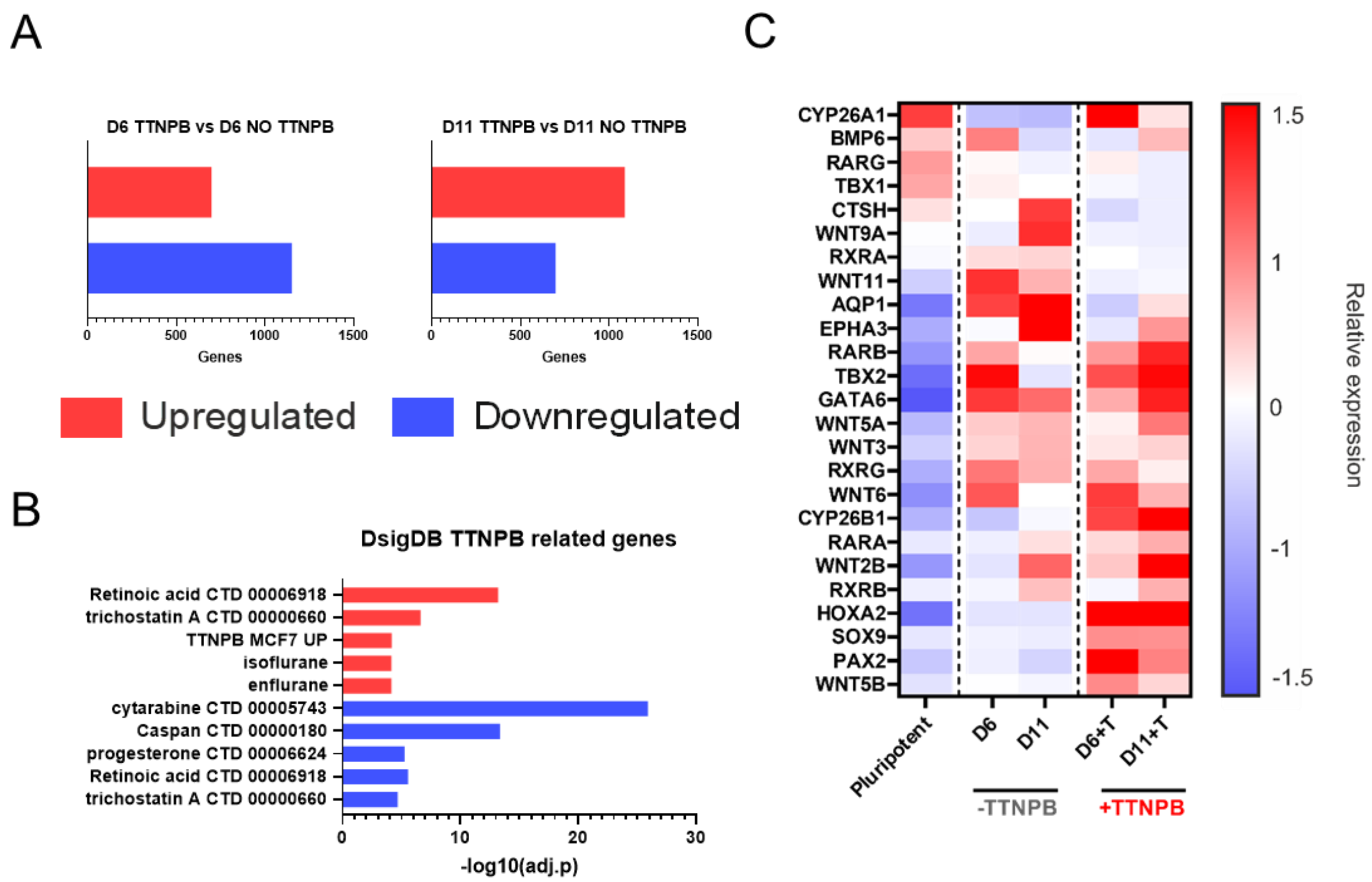

Supplementary Figure 5 –Analysis of transcriptional changes induced by TTNPB addition. (A) Histograms indicating number of differentially expressed genes when comparing Day 6 or Day 11 samples differentiated with or without TTNPB addition. **(B)** Dsig enrichment analysis of common genes between Day 11 and day 6 samples. **(C)** Heatmap illustrating expression profiles of genes regulated by retinoic acid signalling**.** The data are presented as relative expression to the mean, with upregulation represented by red and downregulation by blue. Only genes with an adjusted p-value <0.1 were included in the heatmap.

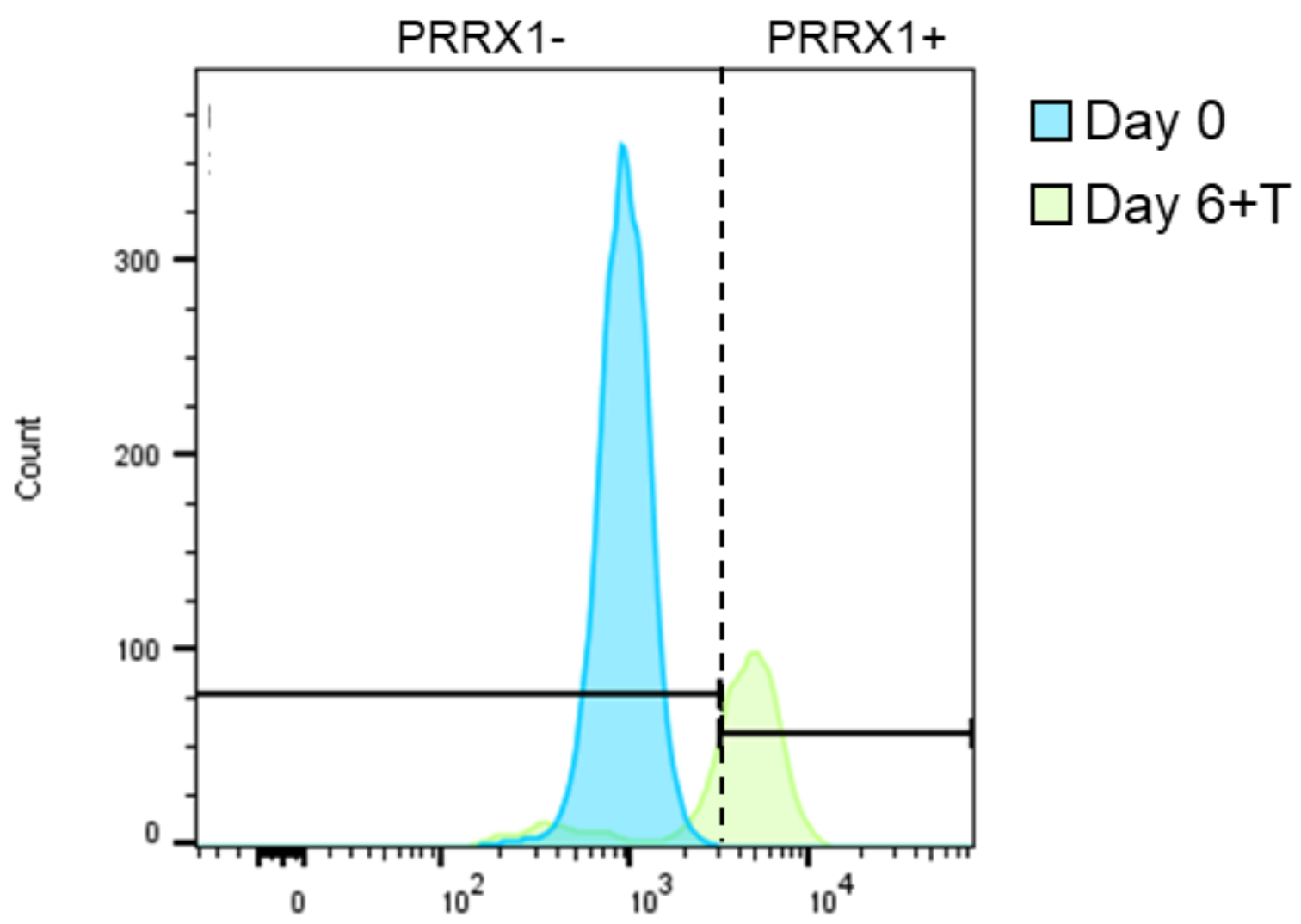

Supplementary Figure 6 - Flow cytometry analysis for the limb-bud mesenchyme marker PRRX1 in MAN13 hESCs differentiated by the RAPID-E protocol. The dotted line represents the gate threshold, dividing the population into positive and negative for the marker considered. Light Blue histogram represent M13 hESCs, the green histogram represent M13 hESCs differentiated by the RAPID-E protocol at day 6.

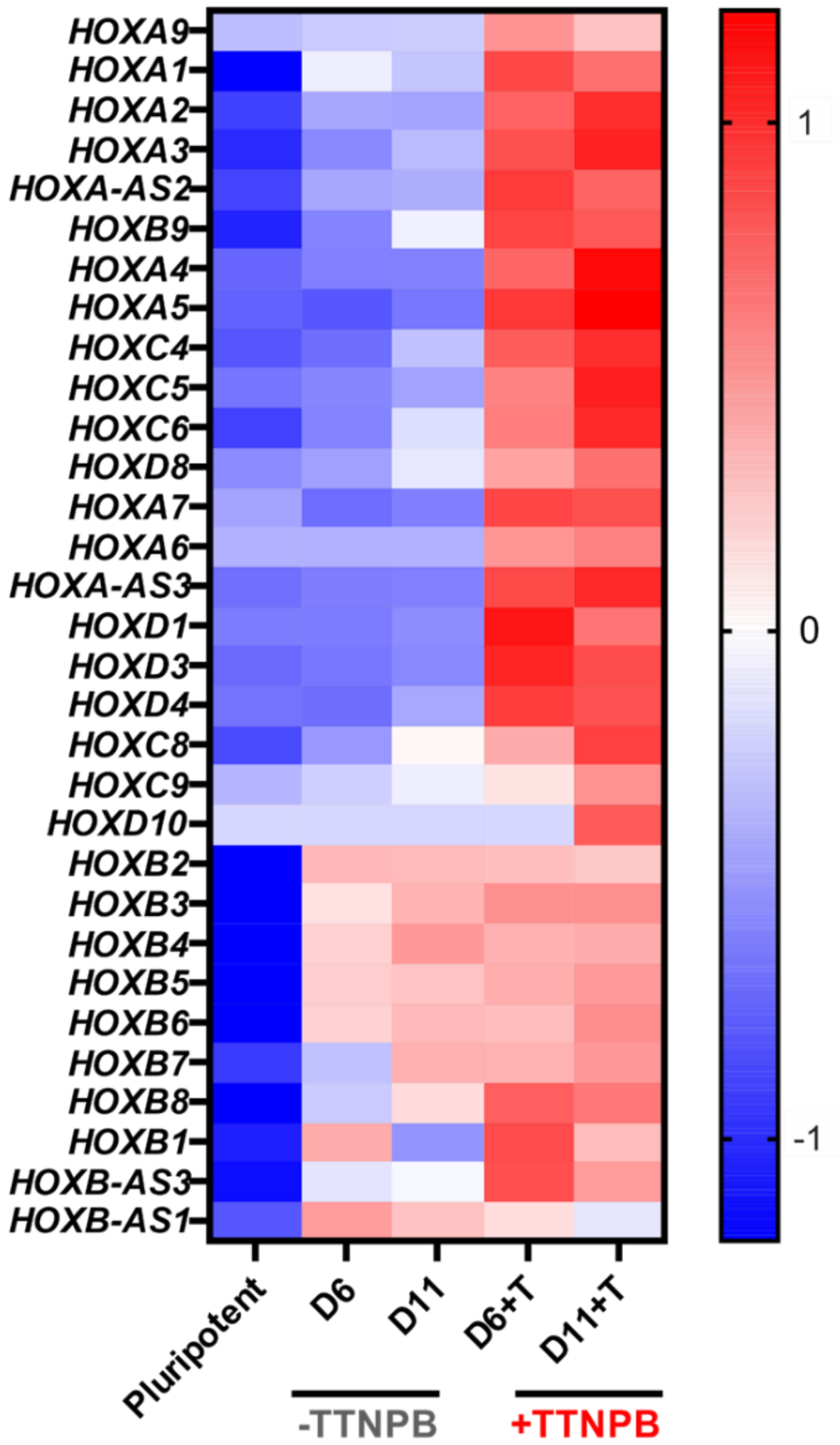

Supplementary Figure 7 - Analysis of HOX gene expression influenced by TTNPB addition. Genes centred using clustering analysis software (31). Gene expression was included only if adjusted p value was <0.1 between 1 or more conditions

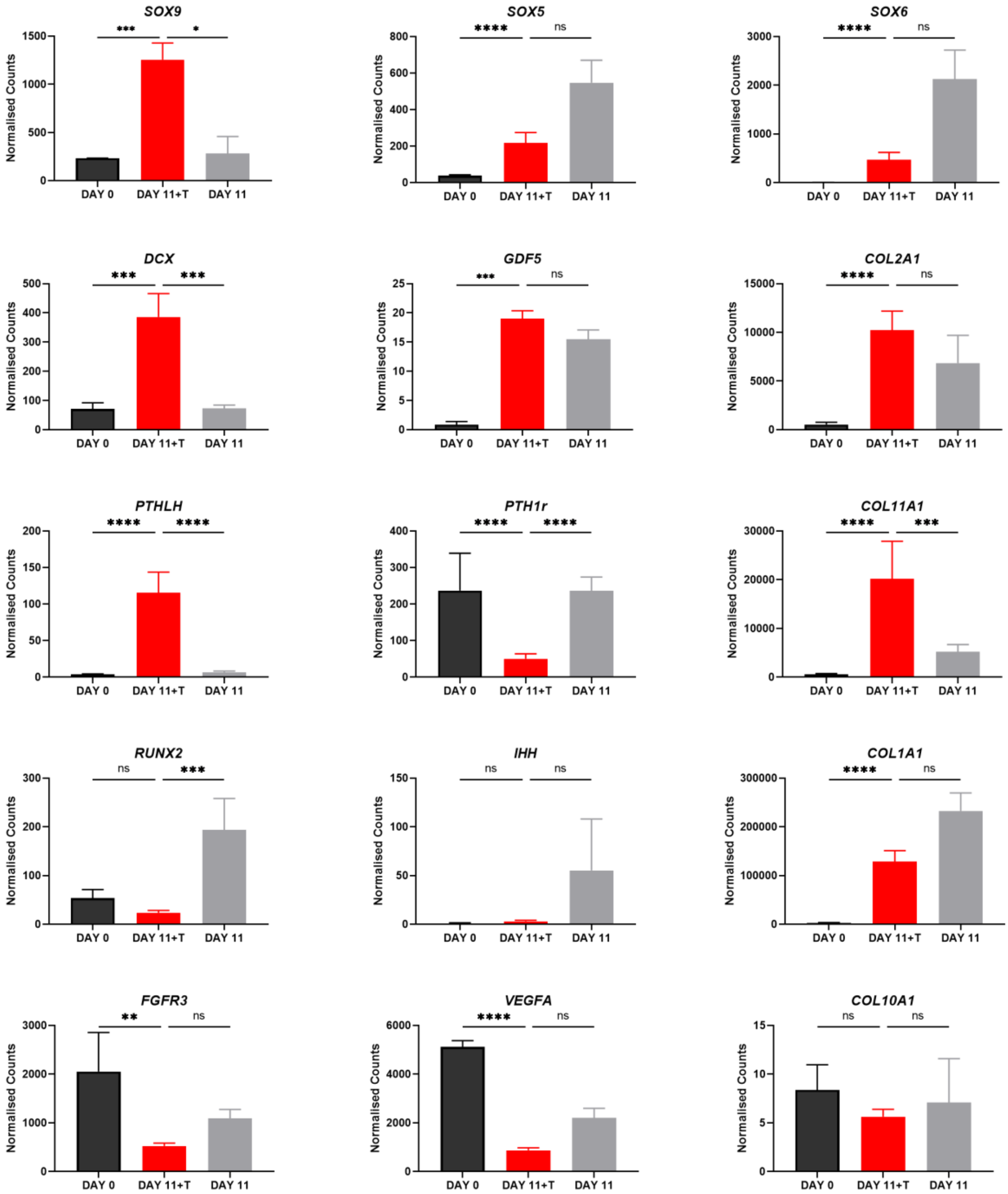

Supplementary Figure 8 - Gene expression of articular and hypertrophic cartilage markers. Data shown as MEAN + SEM of N=3 different biological repeats, normalised read counts were taken from the bulk RNA-seq experiment. Statistical differences between DEGs were calculated using DESeq2 and adjusted p values are shown. (*adj. p<0.1, **adj. p<0.05, ***adj. p<0.005, ****adj. p<0.001).

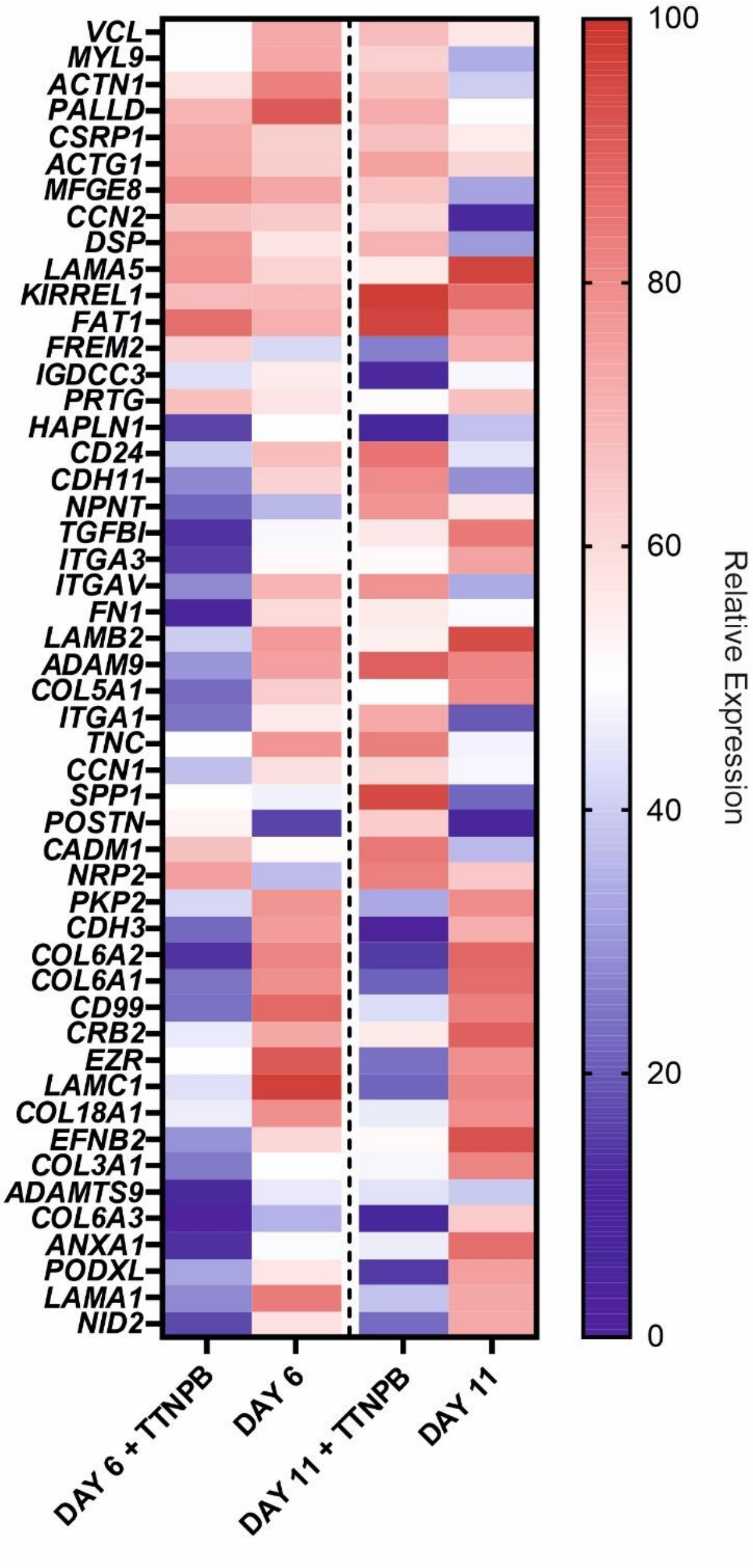

Supplementary Figure 9 – Analysis of top 50 most expressed genes associated with "Cell adhesion" (GO:0098602) at day 6 and day 11. Genes centred using clustering analysis software (31).

Supplemental Experimental Procedures

Supplementary Table 1 – Overview of the RAPID-E differentiation protocol – For allowing pellet formation, TTNPB supplementation was ceased at day 10.

**STAGE**	**Mid-primitive streak**	**Lateral plate mesoderm**	**Limb Bud Mesoderm**	**Chondrogenesis**			**3D Chondrogenesis**	**Manufacturer**
**DAY**	1-2	3-4	5-6	7-8	9	10-15	15-28	
**Activin A**	10 ng/ml							Peprotech, #120-14
**BMP2**					50ng/ml	50ng/ml	25ng/ml	Peprotech, #120-02
**CHIR**	3µM							Tocris, #4423
**FGF2**	20 ng/ml	10ng/ml	10ng/ml	10ng/ml				Peprotech, #100-18B
**PIK90**	100nM							Selleckchem, #S1187
**C59**		1µM		1µM				Abcam, #ab142216
**SB431542**		2µM						Tocris, #1614
**GDF5**				20ng/ml	40ng/ml	40ng/ml	20ng/ml	Peprotech, #120-01
**TGFβ3**							10ng/ml	Peprotech, #100-36E
**TTNPB**	100nM	100nM	100nM	100nM	100nM			Tocris #0761
**SUBSTRATE**	Vitronectin		Fibronectin					
**MEDIUM**	ADBM						ADBM + A2P	

Supplementary Table 2 - Overview of antibodies used for immunohistochemistry and the concentration used.

**Target**	**Isotype**	**Dilution**	**Product code**	**Secondary antibody**
Aggrecan	Rabbit Polyclonal	1:200	Kind Gift Prof. T Hardingham (92)	Goat anti-rabbit
Collagen I	Mouse IgG	1:500	Abcam #ab6308	Horse anti-mouse
Collagen II	Mouse IgG	1:200	Abcam #ab185430	Horse anti-mouse
Collagen X	Rabbit IgG	1:200	Abcam #ab58632	Goat anti-rabbit
Lubricin	Rabbit IgG	1:200	Abcam #ab94933	Goat anti-rabbit
SOX9	Mouse IgG	1:100	Abcam #76997	Horse anti-mouse

Supplementary Table 3 – Primers used for RT-qPCR

Target Gene	Forward (5' to 3')	Reverse (5' to 3')
GAPDH	ATGGGGAAGGTGAAGGTCG	TAAAAGCAGCCCTGGTGACC
POU5F	AGACCATCTGCCGCTTTGAG	GCAAGGGCCGCAGCTTA
NANOG	GGCTCTGTTTTGCTATATCCCCTAA	CATTACGATGCAGCAAATACAAGA
SOX5	ATCCCAACTACCATGGCAGCT	TGCAGTTGGAGTGGGCCTA
SOX9	GACTTCCGCCACGTGGAC	GTTGGGCGGCAGGTACTG
COL1A1	CAGCCGCTTCACCTACAGC	TTTTGTATTCAATCACTGTCTTG
COL2A1	GGCAATAGCAGGTTCACGTACA	CGATAACAGTCTTGCCCCACTT
COL10A1	CAGGCATAAAAGGCCCACTA	AGGACTTCCGTAGCCTGGTT
ACAN	GCAGAGACGCATCTAGAAATTG	GGTAATTGCAGGGAACATCATT
RUNX2	GCCTTCAAGGTGGTAGCCC	CGTTACCCGCCATGACAGTA
PRG4	GAGTACCCAATCAAGGCATTATCA	CCATCTACTGGCTTACCATTGCA
BMPR1B	GAGGATGACTCTGGGTTGCC	AGGCAGTGTAGGGTGTAGGT
FOXF1	AGCAGCCGTATCTGCACCAGAA	CTCCTTTCGGTCACACATGCTG
HAND1	GTGCGTCCTTTAATCCTCTTC	GTGAGAGCAAGCGGAAAAG
HAND2	ACATCGCCTACCTCATGGAC	TTCTTGTCGTTGCTGCTCAC
ISL1	AGATTATATCAGGTTGTACGGGATCA	ACACAGCGGAAACACTCGAT
NKX-2.5	CAAGTGTGCGTCTGCCTTT	CAGCTCTTTCTTTTCGGCTCTA
PRRX1	TGATGCTTTTGTGCGAGAAGA	AGGGAAGCGTTTTTATTGGCT
HOXB5	AACTCCTTCTCGGGGCGTTAT	CATCCCATTGTAATTGTAGCCGT
TBX6	AAGTACCAACCCCGCATACA	TAGGCTGTCACGGAGATGAA
PAX1	CTCACAGCTGGCAGGGTATC	TTAGAGACTGCATGTTAGTTCTGGA
